# A bismuth oxide-modified copper host achieving bubble-free and stable potassium metal batteries†

**DOI:** 10.1039/d4sc07483a

**Published:** 2024-12-12

**Authors:** Guokai Shi, Junpeng Xie, Zhibin Li, Peng Sun, Ying Yin, Likun Pan, Kwun Nam Hui, Wenjie Mai, Jinliang Li

**Affiliations:** a Siyuan Laboratory, Guangdong Provincial Engineering Technology Research Center of Vacuum Coating Technologies and New Energy Materials, College of Physics & Optoelectronic Engineering, Department of Physics, Jinan University Guangzhou 510632 China lijinliang@email.jnu.edu.cn; b Shenzhen Automotive Research Institute, Beijing Institute of Technology Shenzhen 518118 Guangdong China; c Advanced Energy Storage Materials and Technology Research Center, Guangdong-Hong Kong Joint Laboratory for Carbon Neutrality, Jiangmen Laboratory of Carbon Science and Technology Jiangmen 529199 Guangdong Province China; d Shanghai Key Laboratory of Magnetic Resonance, School of Physics and Electronic Science, East China Normal University Shanghai 200241 China; e Joint Key Laboratory of the Ministry of Education, Institute of Applied Physics and Materials Engineering, University of Macau, Avenida da Universidade Taipa Macau SAR 519000 P. R. China

## Abstract

Due to the minimal electrochemical oxidation–reduction potential, the potassium (K) metal anode has emerged as a focal in K-ion batteries. However, the reactivity of the K metal anode leads to significant side reactions, particularly gas evolution. Mitigating gas generation from K metal anodes has been a persistent challenge in the field. In this study, we propose a dual protective layer design through pre-treatment of the K metal anode, employing a Bi_2_O_3_ modification layer alongside a stable solid electrolyte interface (SEI) formed during the initial charge–discharge cycle, which significantly suppresses gas evolution. Furthermore, we observe that the Bi_2_O_3_ modification layer enhances K nucleation due to its strong potassiophilicity when incorporated into the substrate material. The resultant SEI, consisting of dual inorganic layers of Bi–F and K–F formed through the Bi_2_O_3_ modification, effectively mitigates side reactions and gas generation while inhibiting dendrite growth. Utilizing a Cu@BO@K host, we achieve a nucleation overpotential as low as 40 mV, with a stability of 1900 h in a Cu@BO@K‖Cu@BO@K cell and a high average Coulombic efficiency of 99.2% in a Cu@BO@K‖Cu cell at 0.5 mA cm^−2^/0.5 mA h cm^−2^. Additionally, Cu@BO@K‖PTCDA also presents a high reversible capacity of 114 mA g^−1^ at 100 mA g^−1^ after 200 cycles. We believe that this work presents a viable pathway for mitigating side reactions in K metal anodes.

## Abstract 2

The Bi_2_O_3_ modified layer contributes to the formation of a stable solid electrolyte interface during charge–discharge process, which significantly suppresses gas evolution in K-ion batteries.

## Graphical abstract

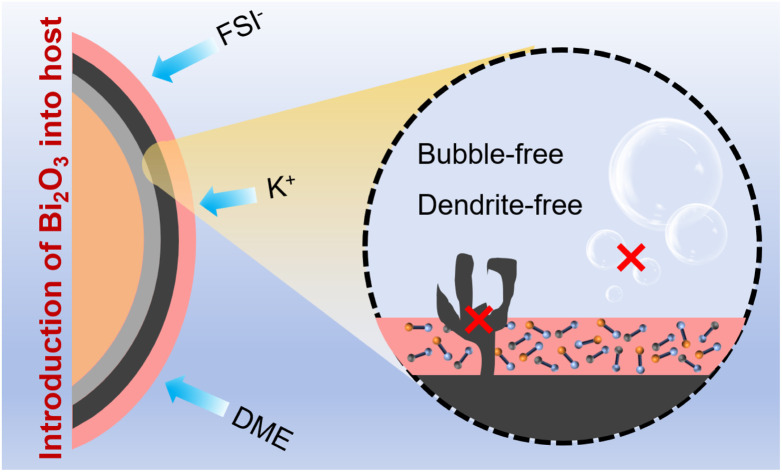

## Introduction

Lithium-ion batteries (LIBs) are favored for energy storage systems due to their long cycle life and high energy density.^1,2^ However, the high cost of lithium resources significantly hinders their further development. As we transition into an era of large-scale energy storage, the focus has shifted toward developing low-cost and highly stable storage technologies.^3–5^ Potassium-ion batteries (KIBs) present notable advantages over traditional LIBs, such as material abundance, affordability, and ionic conductivity, making them promising candidates for cost-effective large-scale energy systems.^6–8^ The choice of electrode materials is vital for achieving high energy density and cycling stability. Metallic K exhibits the highest theoretical capacity (687 mA h g^−1^) among K-ion carriers, leading to increased interest in K metal anodes for higher energy density outputs.^9,10^ However, like many metal anode systems, K metal batteries (KMBs) face critical challenges, particularly dendrite growth and severe side reactions that limit their practical applications.^11–13^

Current strategies to suppress dendrite formation largely involve improving the interface or materials of current collectors to enhance their potassiophilicity.^14^ Previous modifications included constructing SnO_2_-coated conductive porous carbon nanofiber frameworks,^15^ applying Al powder or graphene coatings,^16^ functionalizing Cu mesh into Cu_3_Pt alloys^17^ and building electrode skins.^18^ These enhancements have successfully improved the potassiophilicity of current collectors and significantly increased the lifespan of symmetric cells. Yet, the high reactivity of metallic K can lead to inefficient plating and stripping, resulting in substantial side reactions.^19,20^ Moreover, conventional current collectors used in KMBs are susceptible to corrosion and gas evolution during prolonged cycling, which poses safety risks to battery systems.^21^

To address the side reactions associated with K metal anodes, researchers have begun to manipulate the K interface by introducing protective layers derived from SbF_3_ or red phosphorus and regulating artificial SEI layers to minimize direct contact between the electrolyte and K metal.^22,23^ While these approaches have shown improvements in Coulombic efficiency (CE) and cycling performance, the substantial expansion characteristics of K metal anodes and the complex working environment can disrupt these modifications. Additionally, artificial SEI layers can change their composition and structure due to battery operation, making it challenging to create dense and uniform stability layers of K metal anodes.^24,25^ This situation allows electrolytes to continue infiltrating the K metal anodes, exacerbating gas generation issues, which can lead to swelling and safety concerns during operation.^21,26^

We propose that in designing high-performance K metal anodes, it is crucial not only to address dendrite growth and reversibility but also to effectively suppress gas generation. This aspect is often overlooked in prior studies. Due to the preferential reaction of Bi_2_O_3_ with K-ions, it readily forms alloys and corresponding oxides. The resulting active layer can effectively isolate the solvent in the electrolyte from the metallic K, thus holding potential to solve the gas evolution issue in KMBs. Therefore, we utilized a 3D Cu host and applied electroplating followed by oxidation to achieve the tight adhesion of Bi_2_O_3_ on the Cu surface (Cu@BO), resulting in electrodes with high potassiophilicity. We found that the Bi_2_O_3_ layer enhances the potassiophilicity on the Cu@BO host due to the lower Fermi level and the presence of Bi and O dual-K-affine sites in Cu@BO. This result promotes uniform nucleation, suppressing dendrite growth in K metal anodes. Based on this design, the Cu@BO host achieved an ultra-stable reversible cycling of 1900 h in Cu@BO@K‖Cu@BO@K symmetric cells while maintaining an exceptional average CE of 99.3% at 0.5 mA cm^−2^/0.5 mA h cm^−2^. Notably, while previous K metal anodes commonly exhibited significant gas evolution, the use of the Cu@BO host in KMBs resulted in almost no gas formation. We attribute this to the spontaneous formation of KF and BiF during K plating, which creates a denser layer that effectively isolates the electrolyte from the K metal, thereby mitigating gas generation.

## Results and discussion

**Fig. 1:**
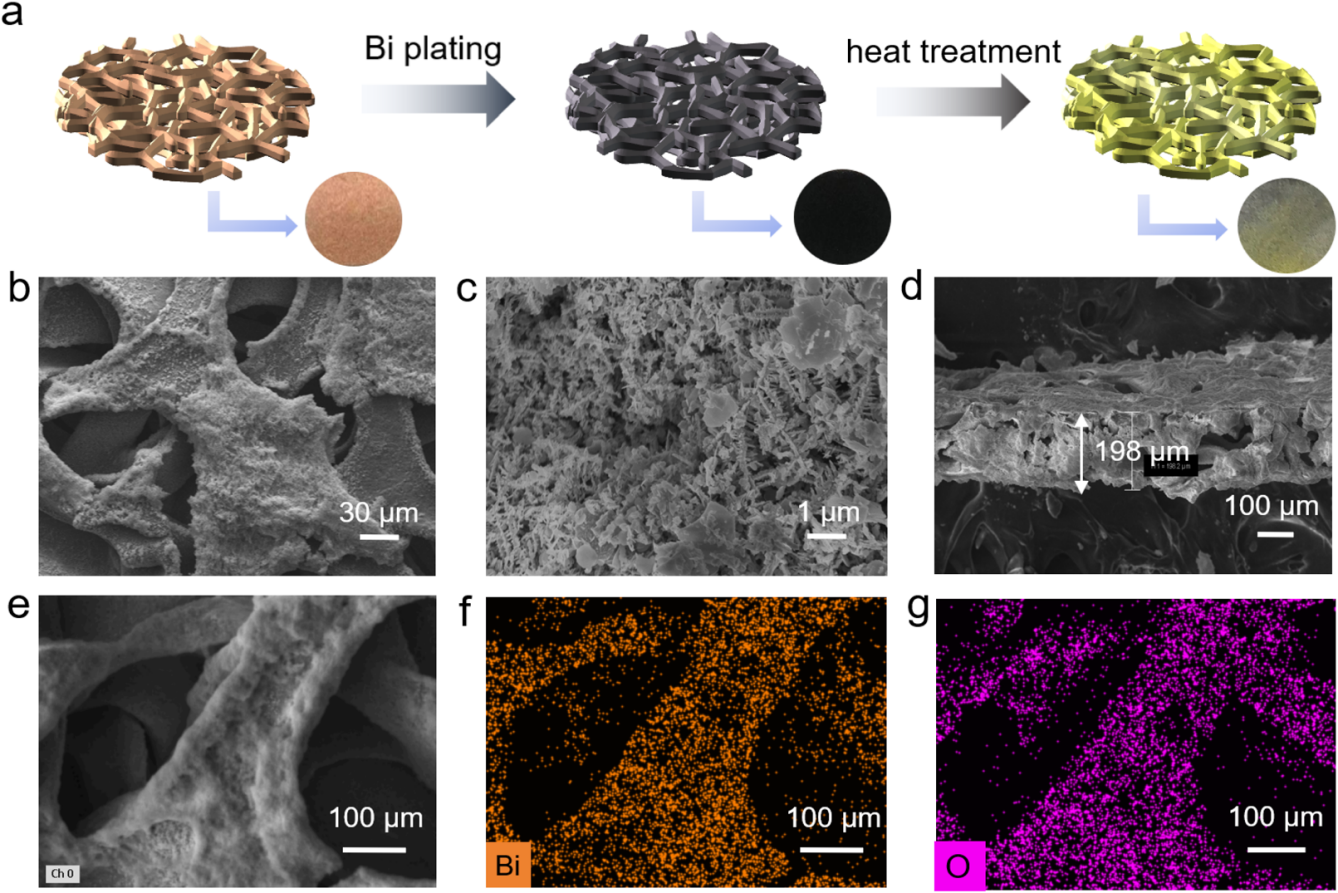
The preparation process and structure information of a Cu@BO host. (a) Preparation process schematic of the Cu@BO host, (b) large-scale, (c) enlarged and (d) cross-sectional SEM images of the Cu@BO host; (e) SEM image and the corresponding (f) Bi and (g) O element mappings of the Cu@BO host.

We obtained the Cu@BO host through electrochemical pre-plating followed by high-temperature oxidation in air. A Cu foam was immersed in a bismuth nitrate aqueous solution and subjected to a fixed voltage. Under the influence of the electric field, metallic Bi was uniformly deposited onto the Cu foam, resulting in Cu@Bi. After washing and drying, the sample underwent high-temperature oxidation in air to yield the Cu@BO host. The detailed fabrication process is illustrated in Fig. 1a. Fig. S1a and b† display the X-ray diffraction (XRD) pattern of Cu@BO, revealing strong diffraction peaks corresponding to Cu (PDF#04-0836) and Bi_2_O_3_ (PDF#27-0050), confirming the substantial presence of Bi_2_O_3_ in the Cu@BO host.^27,28^ The Raman spectrum of Cu@BO is shown in Fig. S1c,† where four peaks at 92, 125, 316, and 466 cm^−1^ correspond to the Bi–O bending vibration, Bi–O–Bi bending vibration, Bi–O stretching vibrations, and Bi–O symmetric stretching, indicating the existence of β-Bi_2_O_3_.^29^ To further investigate the loading of Bi_2_O_3_ on the Cu@BO host, we performed scanning electron microscopy (SEM) analysis. Fig. S2a and b† present the surface of Cu foam, characterized by a smooth interface. After the deposition of metallic Bi, the Cu@Bi surface exhibited distinct fibrous attachments (Fig. S2c and d†). Following oxidation to form Cu@BO, Bi_2_O_3_ remained visibly attached to the surface of Cu foam (Fig. 1b). Although some structural degradation was observed in the magnified image of Cu@BO compared with Cu@Bi, fibrous features of Bi_2_O_3_ were still clearly discernible. Fig. 1d shows a cross-sectional view of the Cu@BO host, revealing a host thickness of approximately 198 μm with a 3D porous morphology. We also conducted element mapping analysis of the Cu@BO host (Fig. 1e and f), which indicated the uniform distribution of Bi and O across the foam surface of Cu foam, confirming the even dispersion of Bi_2_O_3_. To further analyze the structure of Bi_2_O_3_ in the Cu@BO substrate, we scraped off Bi_2_O_3_ and performed transmission electron microscopy (TEM). The needle-like structure of Bi_2_O_3_ can be observed in Fig. S3a.† From the high resolution TEM image in Fig. S3b,† clear lattice fringes were observed with a lattice spacing of 0.328 nm, corresponding to the (201) plane of β-Bi_2_O_3_, consistent with the XRD results.^30,31^

**Fig. 2:**
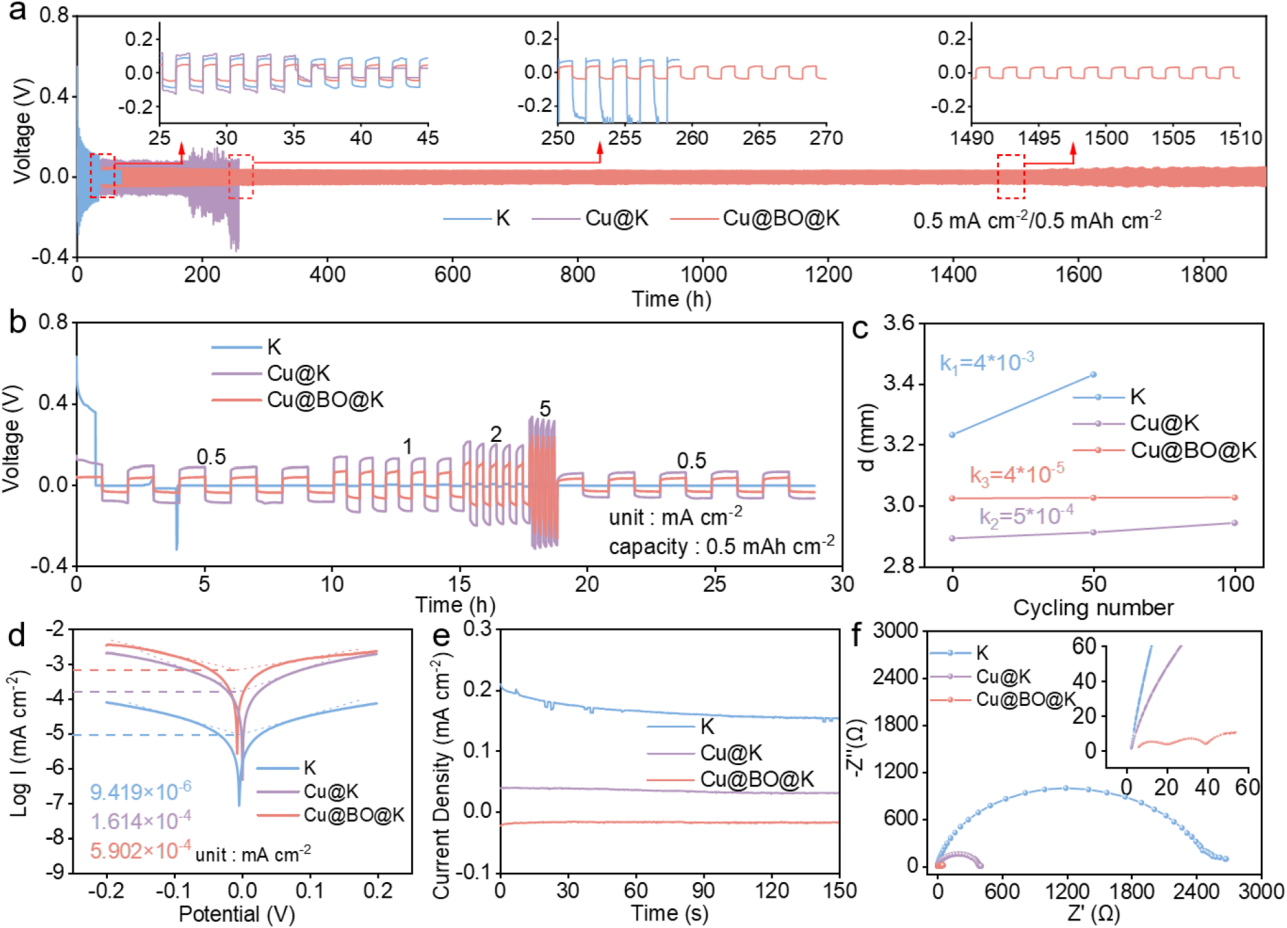
Electrochemical testing of symmetric cells. (a) Cycling and (b) rate performances of bare K‖bare K, Cu@K‖Cu@K and Cu@BO@K‖Cu@BO@K cells at 0.5 mA cm^−2^/0.5 mA h cm^−2^, and the insets in (a) show enlarged charge–discharge curves at 35 h, 260 h and 1500 h. (c) Thickness of the bare K‖bare K, Cu@K‖Cu@K and Cu@BO@K‖Cu@BO@K cells after cycles at 0.5 mA cm^−2^/0.5 mA h cm^−2^. (d) Tafel plots, (e) CA and (f) EIS curves of bare K‖bare K, Cu@K‖Cu@K and Cu@BO@K‖Cu@BO@K cells.

To investigate the potassiophilicity of Cu@BO, we brought it into contact with molten K and observed rapid adsorption of the molten K within 5 s (Fig. S4†). In contrast, the Cu electrode failed to adsorb molten K, confirming that Bi_2_O_3_ enhances the potassiophilicity of the Cu@BO host. Building on this property, we electrochemically deposited a certain amount of metallic K onto the Cu@BO electrode (denoted as Cu@BO@K), while also depositing metallic K onto the Cu foam electrode (Cu@K) for comparison, using bare K as a control. The electrolyte and separator used were 5 m potassium bis(fluorosulfonyl)imide in dimethoxyethane (DME) and glass fiber, respectively. Fig. 2a presents the cycling performance of the bare K‖bare K, Cu@K‖Cu@K, and Cu@BO@K‖Cu@BO@K symmetric cells. It is found that the bare K‖bare K cell experienced a short circuit after less than 100 h of cycling due to K dendrite growth piercing the separator at 0.5 mA cm^−2^/0.5 mA h cm^−2^. The Cu@K‖Cu@K cell exhibited a polarization voltage of 200 mV, which increased over 200 h at 0.5 mA cm^−2^/0.5 mA h cm^−2^, indicating significant side reactions on the Cu@K surface and notable interface degradation. In contrast, the Cu@BO@K‖Cu@BO@K cell displayed markedly enhanced stability, maintaining a low polarization voltage of 52 mV even after 1500 h at 0.5 mA cm^−2^/0.5 mA h cm^−2^, suggesting that the potassiophilicity of the oxygen-doped structure reduces the nucleation overpotential for K ions and highlights the synergistic effect of the active Bi and O sites.^32^Fig. 2b shows the cycling performance of the three different symmetric cells at varying current densities (0.5–5 mA cm^−2^/0.5 mA h cm^−2^). The bare K‖bare K cell could not complete the testing due to rapid short-circuiting during the rate testing. Compared to the Cu@K‖Cu@K cell, the Cu@BO@K‖Cu@BO@K cell exhibited distinct reduced polarization voltages, demonstrating that the introduction of Bi_2_O_3_ in Cu@BO enhances the deposition capability of K ions on the host.^33^ From the symmetric cell, we also observed a degree of swelling in both the bare K‖bare K and Cu@K‖Cu@K cells while the Cu@BO@K‖Cu@BO@K cell shows little of this swelling. We attribute this swelling in bare K‖bare K and Cu@K‖Cu@K cells to side reactions occurring within the cells. To quantify this phenomenon, we measured the thickness of the bare K‖bare K, Cu@K‖Cu@K and Cu@BO@K‖Cu@BO@K cells after different cycles, as shown in Fig. 2c. The bare K‖bare K cell showed the largest thickness change compared with the initial state with increased cycling, while the thickness change in Cu@K‖Cu@K was reduced, indicating mitigation of side reactions. In contrast, the Cu@BO@K‖Cu@BO@K cell exhibited minimal change in thickness, confirming that side reactions within the cell were significantly suppressed.

Fig. 2d presents the Tafel curves for bare K, Cu@K, and Cu@BO@K in symmetric cells. It is found that Cu@BO@K presents an exchange current density of 5.902 × 10^−4^ mA cm^−2^, which exhibits higher exchange current density compared to bare K (1.614 × 10^−4^ mA cm^−2^) and Cu@K (9.419 × 10^−6^ mA cm^−2^), indicating superior kinetics for Cu@BO@K in the K metal anode.^34,35^ The chronoamperometry (CA) tests for all the symmetric cells are illustrated in Fig. 2e. The CA results indicate that the current density at a constant potential sensitively reflects the nucleation process and surface changes. All the CA tests operated at a constant voltage of −10 mV. An oblique CA curve can be observed in the bare K‖bare K cell and this process was maintained approximately for 45 s. This process indicates 2D diffusion during the K plating. After 45 s, the CA curve of the bare K‖bare K cell turned to almost constant current, indicating the K plating process transitioning to 3D diffusion.^36^ Compared with the bare K‖bare K cell, the current for Cu@K‖Cu@K and Cu@BO@K‖Cu@BO@K cells showed little variation, indicating that their 2D diffusion is suppressed. However, the Cu@BO@K‖Cu@BO@K cell exhibited a lower polarization current density of 0.018 mA cm^−2^, which is lower than that of the Cu@K‖Cu@K cell (0.03 mA cm^−2^). Low polarization current density reflects a stable nucleation process governed by 3D diffusion.^37^ To evaluate the electronic and ionic transfer capabilities of our cells, we also conducted electrochemical impedance spectroscopy (EIS) of the bare K‖bare K, Cu@K‖Cu@K and Cu@BO@K‖Cu@BO@K cells after cycling. Fig. S5† shows the corresponding equivalent circuit diagram. The results revealed that the Cu@BO@K‖Cu@BO@K cell exhibited significantly lower impedance (*R*_ct_ = 40 Ω) compared to the Cu@K‖Cu@K cell (*R*_ct_ = 405 Ω) and bare K‖bare K cell (*R*_ct_ = 2678 Ω) after fitting, further confirming the faster kinetics of Cu@BO@K. The pre-treated Bi_2_O_3_ modification layer contributes to the formation of a more effective solid electrolyte interphase (SEI) layer, facilitating ion transport.

**Fig. 3:**
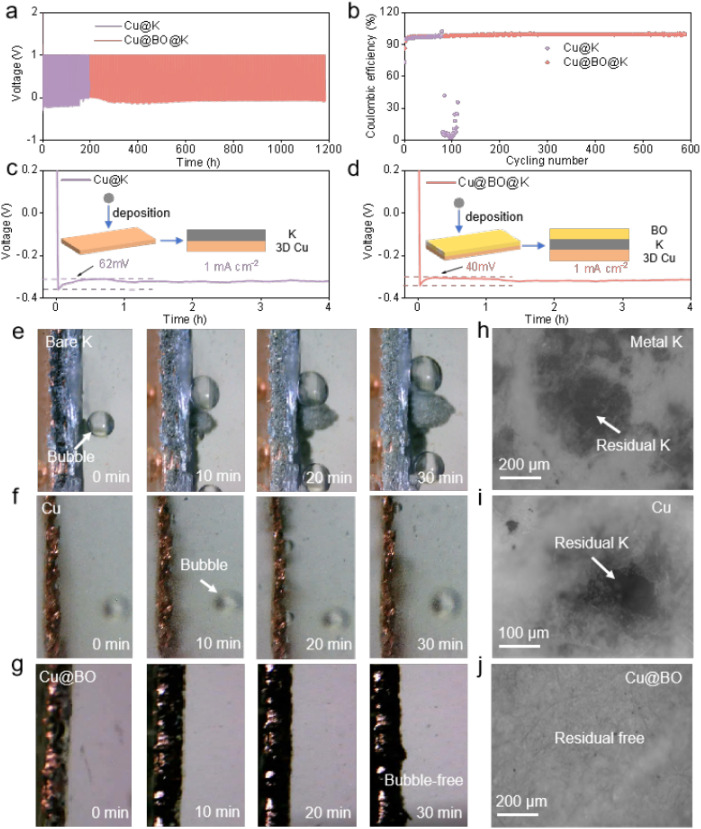
Reversibility of the K metal anode. (a) Voltage profiles and (b) CEs of Cu@K‖Cu and Cu@BO@K‖Cu cells at 0.5 mA cm^−2^/0.5 mA h cm^−2^. (c) Voltage profiles during initial K plating on (c) Cu@K‖Cu and (d) Cu@BO@K‖Cu host at 0.5 mA cm^−2^; the insets show the K deposition schematic. *In situ* optical microscope photographs for (e) bare K, (f) Cu and (g) Cu@BO hosts at a plating areal current density of 0.5 mA cm^−2^. High resolution optical microscope images of separators with (h) bare K, (f) Cu and (g) Cu@BO hosts after 100 cycles at 0.5 mA cm^−2^/0.5 mA h cm^−2^.

To further evaluate the reversibility of Cu@K and Cu@BO@K in battery applications, we assembled Cu@K‖Cu and Cu@BO@K‖Cu asymmetric cells to test their electrochemical performance. Fig. 3a presents the charge–discharge curves of these asymmetric cells at 0.5 mA g^−1^/0.5 mA h g^−1^ and Fig. S6† shows the enlarged charge–discharge curves of these asymmetric cells. The Cu@K‖Cu cell demonstrated a limited cycling capability, achieving only 110 cycles (220 h), while the Cu@BO@K‖Cu cell exhibited a substantial increase in cycling life, achieving 598 cycles (1196 h). Furthermore, the CE of the Cu@K‖Cu half-cell dropped sharply after 110 cycles, indicating significant irreversible phenomena at the metallic K anode interface. In contrast, the Cu@BO@K‖Cu cell maintained an impressive average CE of 99.2% over 598 cycles, confirming that the addition of Bi_2_O_3_ effectively enhances the deposition and stripping of metallic K (Fig. 3b). To investigate the underlying reasons for the enhanced reversibility in the Cu@BO@K cell, we compared the voltage–time profiles of Cu@K and Cu@BO@K at a current density of 1 mA cm^−2^ (Fig. 3c and d). Both cells exhibited a rapid voltage drop during the initial deposition phase, indicative of 2D diffusion of metallic K. Subsequently, the voltage curves flattened, signaling a transition to diffusion.^38^ Notably, the Cu@K‖Cu cell transitioned to the 3D diffusion region after 35 min, whereas the Cu@BO@K‖Cu cell entered this region after just 5 min, demonstrating accelerated kinetics under the Cu@BO@K substrate. We also compared the overpotentials of the Cu@K‖Cu and Cu@BO@K‖Cu cells, finding that the overpotential for the Cu@BO@K‖Cu cell (40 mV) was significantly lower than that for Cu@K‖Cu (62 mV). This further substantiates that metallic K nucleates more readily on the Cu@BO@K host, promoting the kinetics of K ion deposition. Fig. S7† presents the CV curves for the Cu@K‖Cu, and Cu@BO@K‖Cu cells. The results reveal that the cell with the Cu@BO host exhibits superior redox properties compared to those with the Cu and K hosts, indicating that the Cu@BO host significantly enhances the reversibility of the K-ion stripping process. To study the deposition state and side reactions of metallic K on different hosts, we constructed a sealed visualization device for *in situ* observation of the metallic K plating process on bare K, Cu@K, and Cu@BO@K at 0.5 mA cm^−2^. Fig. 3e shows *in situ* optical photographs of bare K, which displayed typical bubble formation upon contact with the electrolyte, indicating a pronounced gas evolution effect. As plating continued, more bubbles appeared on the bare K surface, confirming persistent gas evolution throughout the plating process. Additionally, notable uneven growth of metallic K led to severe dendrite formation, which can induce short circuits, consistent with rapid short-circuiting observed in symmetric cells. When using Cu@K as the substrate, no significant dendrite formation was observed, suggesting that the Cu@K electrode partially mitigates the dendrite issue. However, gas bubbles persisted in the electrolyte, indicating that encapsulating metallic K does not effectively resolve the gas evolution problem. The presence of gas evolution can lead to ongoing decomposition of the electrolyte during subsequent cycling, exacerbating interfacial degradation and increasing polarization. In comparison to bare K and Cu@K electrodes, Fig. 3g illustrates that no dendrites formed during cycling on the Cu@BO@K electrode, indicating that the 3D structure of Cu foam helps suppress rapid dendrite growth. Notably, no gas bubbles were observed in the electrolyte during the plating process with the Cu@BO@K electrode, confirming that the introduction of Bi_2_O_3_ effectively isolates metallic K from the electrolyte, significantly reducing the gas evolution effect.

**Fig. 4:**
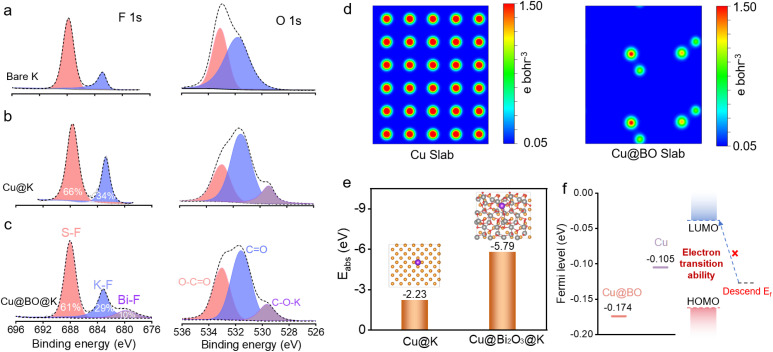
XPS spectra and theoretical calculation of the hosts. High resolution F 1s and O 1s XPS of (a) bare K, (b) Cu@K and (c) Cu@BO@K host after cycles. (d) Surface charge density and (e) surface adsorption energy of Cu@K and Cu@BO@K. (f) Plots of the Fermi levels of Cu and Cu@BO host to the electrolyte.

To further confirm the suppression of gas bubble formation on the Cu@BO@K electrode, we provided *in situ* optical photographs of K plating on both Cu and Cu@BO electrodes at high current density (Fig. S8†). Compared to the Cu electrode, the Cu@BO electrode remained bubble-free even at 10 mA g^−1^, further demonstrating that Bi_2_O_3_ in the electrode effectively inhibits gas evolution resulting from electrolyte decomposition. Given that this observation captures only the initial stages, it cannot fully elucidate the side reactions throughout the entire cycling period. We also present high-resolution optical microscopy images of the separators from the symmetric cells after 200 h of cycling with bare K (Fig. 3h), Cu@K (Fig. 3i), and Cu@BO@K (Fig. 3j). The separator from the bare K cell exhibited a significant accumulation of black dead K, indicative of an unstable SEI formation, which promotes corrosion of the metallic K and leads to dead K formation. Similarly, residual K was observed on the Cu@K separator due to continuous gas evolution side reactions causing detachment of metallic K; however, the interface showed some improvement compared to bare K. In stark contrast, the separator from the Cu@BO@K cell showed minimal residual dead K, further confirming that Bi_2_O_3_ significantly inhibits side reactions involving metallic K. We also provide the photographs of Cu@K and Cu@BO@K electrodes after plating, as shown in Fig. S9a and b.† It is found that the K metal on the Cu electrode appears white and uneven, while the K metal on the Cu@BO electrode retains its metallic luster, demonstrating that K ions can be uniformly plated on the Cu@BO electrode. In addition, from the high-resolution optical microscopy images of the symmetric cells after 20 h of cycling with Cu@K (Fig. S9c†) and Cu@BO@K (Fig. S9d†), obvious exposed Cu and dendrites can be observed in the Cu host. In contrast, fewer dendrites in Cu@BO@K can be observed, further indicating the Bi_2_O_3_ significantly inhibits the dendrite growth. To further investigate the characteristics of bare K, Cu@K, and Cu@BO@K in battery applications, we conducted X-ray photoelectron spectroscopy (XPS) analysis on different hosts post-cycling, as shown in Fig. 4a–c and S10.† Notably, prominent F 1s peaks were observed in all three hosts. After deconvolution, the F 1s XPS spectra of bare K and Cu@K hosts revealed two distinct peaks at 688.0 eV and 683.2 eV, corresponding to C–F and K–F bonds, respectively.^39,40^ In contrast, the Cu@BO@K host exhibited a new peak at 679.8 eV, attributed to Bi–F bonds. Our findings indicated that the K–F bond content was lowest in bare K, while it significantly increased in Cu@K. Previous studies have established that a high content of K–F bonds is crucial for forming a high-quality SEI layer on K metal anodes and that this enhancement is beneficial for suppressing K dendrite growth.^41,42^ Consequently, the cycling stability of Cu@K was improved relative to bare K. However, while comparing Cu@K to Cu@BO@K, a slight decrease in K–F bond content was observed alongside the emergence of the new Bi–F bond. The presence of these dual inorganic fluorides facilitates the formation of a high-quality SEI layer.^43^ A comparison of the K 2p and C 1s XPS spectra shows minimal shifts in their corresponding peak positions, indicating that the introduction of Bi_2_O_3_ has no significant effect on K ions or organic species.

To further elucidate the role of Bi_2_O_3_ in the K metal anodes, we employed density functional theory (DFT) calculations to investigate the charge distribution in Cu and Cu@BO (Fig. 4d). The results indicated a high electron cloud density around all atomic centers in Cu, whereas the introduction of the Bi_2_O_3_ modification layer in Cu@BO resulted in a decrease in atomic charge distribution. This effect is attributed to the strong electron-withdrawing nature of Bi_2_O_3_, which promotes passivation at the electrode interface.^44^ Additionally, we calculated the adsorption energies of K ions on both Cu and Cu@BO, as illustrated in Fig. 4e. The binding energy between the K atom and Cu was found to be −2.23 eV. With the addition of Bi_2_O_3_, the adsorption energy (*E*_abs_) for K ions on Cu@BO decreased to −5.79 eV, indicating a preference for K to adsorb on the Cu@BO surface.^45,46^ We also compared the Fermi levels of Cu and Cu@BO, revealing a Fermi level of −0.105 eV for Cu, which decreased to −0.174 eV upon Bi_2_O_3_ loading in Cu@BO (Fig. 4f). The lowered Fermi level of the host results in diminished electron transitions within the electrode materials to the lowest unoccupied molecular orbital (LUMO) energy level of DME, thereby inhibiting charge transfer between Cu@BO and the DME solvent.^47^ Given that gas evolution primarily stems from solvent decomposition, suppressing electron transfer from the electrode to the DME electrolyte significantly mitigates the gas evolution effects during the stripping/plating processes of the K metal anode.

**Fig. 5:**
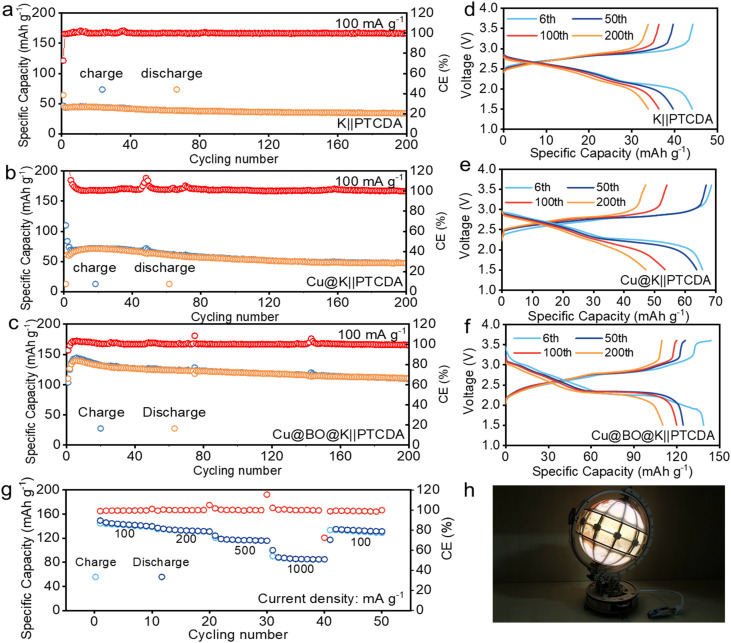
Electrochemical performance of full cells. The cycling performances of (a) PTCDA‖K, (b) PTCDA‖Cu@K, and (c) PTCDA‖Cu@BO@K full cells at 100 mA g^−1^. Corresponding galvanostatic charge–discharge curves of (d) PTCDA‖K, (e) PTCDA‖Cu@K, and (f) PTCDA‖Cu@BO@K full cells. (g) Rate performance of the PTCDA‖Cu@BO@K full cells at different current densities of 100 to 1000 mA g^−1^. (h) PTCDA‖Cu@BO@K full cell powered LED lights.

Building on the advantages of performance and structure, we paired the perylene tetracarboxylic dianhydride (PTCDA) cathode with our K metal anode to assemble full cells for further assessment of the practicality. Fig. 5a–c illustrate the cycling performance of PTCDA‖K, PTCDA‖Cu@K, and PTCDA‖Cu@BO@K cells. Notably, the PTCDA‖K cell exhibited an initial specific capacity of only 45.2 mA h g^−1^, which diminished to 33.9 mA h g^−1^ at 100 mA g^−1^ after 200 cycles. This low capacity is attributed to gas generation at the surface of the K anode, which reduces the effective contact between the electrolyte and the electrode material, hampering K-ion storage efficiency. In comparison, the PTCDA‖Cu@K cell demonstrated an initial specific capacity of 68.3 mA h g^−1^, maintaining this capacity of 47.2 mA h g^−1^ through 200 cycles. This modest improvement can be ascribed to a reduction in gas evolution effects on the electrode surface. However, considering the presence of bubbles in the PTCDA‖Cu@K cell, there is still an issue with the contact between the electrolyte and the electrode, which causes low capacity. When Bi_2_O_3_ was introduced, the gas evolution was significantly suppressed. This behavior results in a sharp reduction of bubbles in the full cell.^48^ The reduction of bubbles can enhance the effective contact between the electrode and electrolyte, thus facilitating efficient ionic exchange. As a result, the PTCDA‖Cu@BO@K full cell achieved a high reversible specific capacity of 140 mA h g^−1^ upon initial discharge and maintained a specific capacity of 112 mA h g^−1^ after 200 cycles. Fig. 5d–f display the corresponding galvanostatic charge–discharge curves of PTCDA‖K, PTCDA‖Cu@K, and PTCDA‖Cu@BO@K full cells. To further investigate the electrochemical characteristics of the PTCDA‖Cu@BO@K cell, we evaluated its rate performance at various current densities. The results showed that the PTCDA‖Cu@BO@K cell presents reversible specific capacities of 141.2, 136.4, 122.8, and 86.7 mA h g^−1^ at 100, 200, 500, and 1000 mA g^−1^, respectively. The PTCDA‖Cu@BO@K full cell also presents a notable capacity recovery when the current density was returned to 100 mA g^−1^. To demonstrate the practical applicability of our full cell, we connected multiple cells in series to power a bright light emitting diode (LED) light (Fig. 5h), affirming the promising application potential of the PTCDA‖Cu@BO@K full cell design.

We can effectively address the gas evolution issue of the K metal anode by tuning the substrate material. However, how to further enhance the electrochemical window remains a key focus in KIB's research. Many researchers have already achieved an improvement in the voltage window of KIBs through electrolyte optimization.^49,50^ In future work, we also hope to combine our Cu@BO host design with high-voltage electrolytes to achieve high energy density output for KMBs.

## Conclusions

In summary, we successfully developed a Cu@BO host by utilizing electrochemical deposition followed by oxidation to load Bi_2_O_3_ onto a Cu substrate. The intrinsic potassiophilicity of Bi_2_O_3_ enables the Cu@BO host to facilitate rapid metallic K adsorption. The introduction of oxygen functional groups and metallic Bi significantly enhances K ion adsorption and precise nucleation during the operation of the K metal anode, while also promoting the spontaneous alloying of Bi_2_O_3_. This results in smooth K ion diffusion and substantially slows down the growth of K dendrites. The Cu@BO@K host exhibits a low nucleation overpotential of just 40 mV and a high average CE of 99.2%, with symmetrical cell stability lasting up to 1900 h at 0.5 mA cm^−2^/0.5 mA h cm^−2^. Furthermore, when assembled into a full cell with a PTCDA cathode, the initial reversible capacity reaches 140 mA h g^−1^ at 100 mA g^−1^. Notably, we observed no gas evolution when employing the Cu@BO host in the K metal anode, indicating that it effectively suppresses gas generation. We attribute this phenomenon to the Bi_2_O_3_ modification layer and the robust SEI membrane featuring Bi–F and K–F bonds, which significantly reduce side reactions between the metallic K and the electrolyte, thus mitigating gas formation. We believe that this work lays a solid foundation for the design of high-performance, gas-free K metal anodes and for exploring and optimizing the application of metallic K electrodes in energy storage technologies.

## Data availability

The data supporting this article have been included as part of the ESI.†

## Author contributions

G. Shi and J. Xie performed investigation, methodology, data curation, wrote the original draft. Z. Li, P. Sun, Y. Yin performed investigation. L. Pan and K. N. Hui performed validation and supervision. W. Mai and J. Li performed supervision, conceptualization and wrote, review & edited the final manuscript.

## Conflicts of interest

There are no conflicts to declare.

## Supplementary Material

SC-016-D4SC07483A-s001
