# Supplementary material for: A bismuth oxide-modified copper host achieving bubble-free and stable potassium metal batteries
Source: Chem Sci. 2024 Dec 12;16(3):1344–52. doi: 10.1039/d4sc07483a (PMC11651390; doi:10.1039/d4sc07483a)
Supplement: SC-016-D4SC07483A-s001 [file SC-016-D4SC07483A-s001.pdf]

Supplementary Information (SI) for Chemical Science.
This journal is © The Royal Society of Chemistry 2024

Supporting Information

Experimental

1. Synthesis

All reagents used in the synthesis were of analytical grade. Typically, Cu foam was cut into 1.5 cm × 1.5 cm pieces and sequentially cleaned in deionized water and ethanol for 10 min each. To prepare the plating solution, 10 mmol of $BiCl_3$ was added to 260 mL of deionized water and 40 mL of concentrated hydrochloric acid (37 wt%) and stirred until clear. A 25 mL portion of this solution was then used for the plating process, where metallic Bi was deposited onto the Cu foam surface using a two-electrode configuration under a constant voltage of 3 V, with Cu foam as the cathode and Pt foil as the anode. Following electroplating, the Cu@Bi was annealed at 200 °C for 2 h in a muffle oven to obtain Cu@BO.

For electrochemical performance testing, the Cu foam and Cu@BO was cut into 14 mm diameter discs, and half-cells were assembled using a 5 M potassium bis(fluorosulfonyl)imide dissolve into dimethoxyethane (DME) as electrolyte. The Cu@BO electrodes were pre-deposited with 5 mAh of metallic K at a current density of 1 mA cm^{-2} to form Cu@BO@K. The uncoated Cu foam electrode was designated as Cu, and the Cu electrode pre-deposited with metallic K using the same method was designated as Cu@K.

For the electrochemical performance testing of full cells, perylene tetracarboxylic dianhydride (PTCDA) cathode, Super P, and polyvinylidene fluoride (PVDF) were mixed in a ratio of 8:1:1. Then, a suitable amount of 1-methyl-2-pyrrolidone was added to form a uniform slurry. The slurry was then coated onto an Al-C foil. After drying, the resulting electrode was cut into 14 mm diameter discs for full-cell assembly.

2. Characterization

Morphological analyses of the electrodes were conducted using scanning electron microscopy (SEM, FEI), transmission electron microscopy (TEM, JEOL), and optical

microscopy (Leica). Structural and surface properties were measured with X-ray diffraction (XRD, Rigaku D8-9000), Raman spectroscopy (Horiba T6400), and X-ray photoelectron spectroscopy (XPS, Thermo Scientific). For *in situ* visualization, a portable optical microscope was used in combination with an electrochemical reaction cell, allowing in situ observation of the electrodes. During open-cell observations, the cell was charged and discharged at a current density of 0.5 mA cm^{-2}.

3. Electrochemical measurement

The electrochemical performance of all samples was evaluated using CR2032 coin cells, which were assembled in an argon-filled glovebox (Etelus Lab2000, O_2 <0.1 ppm, H_2O <0.1 ppm). The electrolyte consisted of 5 M potassium bis(fluorosulfonyl)imide dissolved in DME. Symmetric cells were constructed with identical electrodes at both ends, while asymmetric cells were assembled with Cu foil as the counter electrode and bare K, Cu@K, or Cu@BO@K as the working electrodes, resulting in bare K||Cu, Cu@K||Cu, and Cu@BO@K||Cu configurations. Full cells were assembled using a PTCDA cathode paired with bare K or Cu@K as the anode. Galvanostatic charge-discharge (GCD) curves were obtained with a Neware battery testing system (Neware BTS-4000), while a CHI electrochemical workstation recorded electrochemical impedance spectroscopy (EIS) and nucleation overpotentials for the symmetric cells.

4. DFT computational methods

The calculation related to the interaction between various crystal slab and K+ were performed by using the Vienna Ab Initio Simulation Package (VASP). The projector augmented wave (PAW) potential was employed to represent the interactions of electrons with ion cores. The generalized gradient approximation (GGA) parameterized by the Perdew-Burke-Ernzerhof (PBE) method with D3 correction was used to describe the system[1].

All calculations including geometry optimization, single-point energy and electronic density were carried out within a 13.2082 × 15.5079 × 34.3344 Å3 box under

a periodic boundary condition and a dense Monkhorst-Pack k point mesh of 2 × 2 × 1. A high energy cutoff of 500 eV and the forces acting on all the ions smaller than 0.03 eV Å−1 were set. On the Z direction, there is about 20 Å vacuum for erasing the effect of periodic condition for slab model. A 6×5 supercell with four-layer Cu slab (200) and Cu (200)-Bi_2O_3 (201) composite slab were used to represent the absorbed surface for molecules and the bottom two layers were kept fixed to maintain bulk property[2].

In details, the Fermi levels was obtained from the single-point energy results of optimized geometries of Cu, Cu-Bi_2O_3 slabs and the DME molecule with a higher energy convergence precision of 10^{-7} eV. The surface charge density was derived by the Electron Localization Function analysis and presented *via* the software Vesta.The absorbed energy between Cu, Cu-Bi_2O_3 slabs and K^+ was defined as following equation:

$$E_{aborb.} = E_{slab+K^+} - E_{slab} - E_{K^+}$$

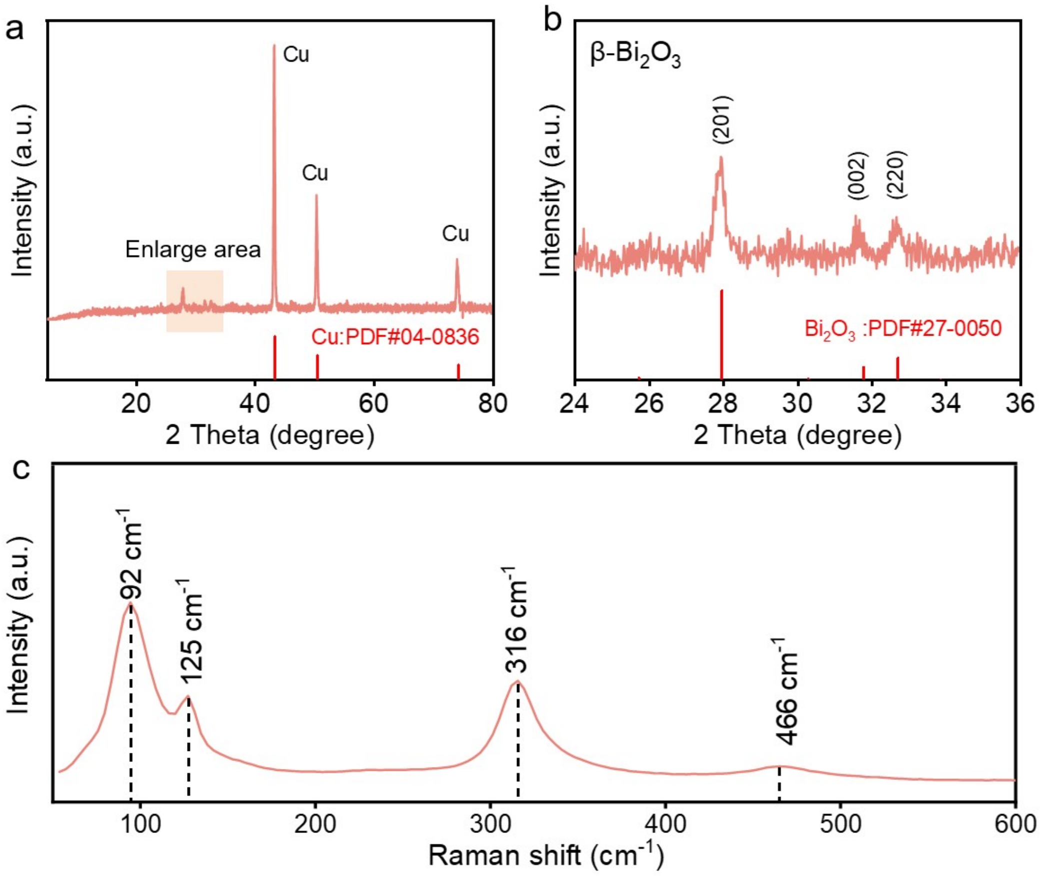

Figure S1 Typical (a) XRD pattern, enlarged XRD pattern and Raman spectrum of the Cu@BO

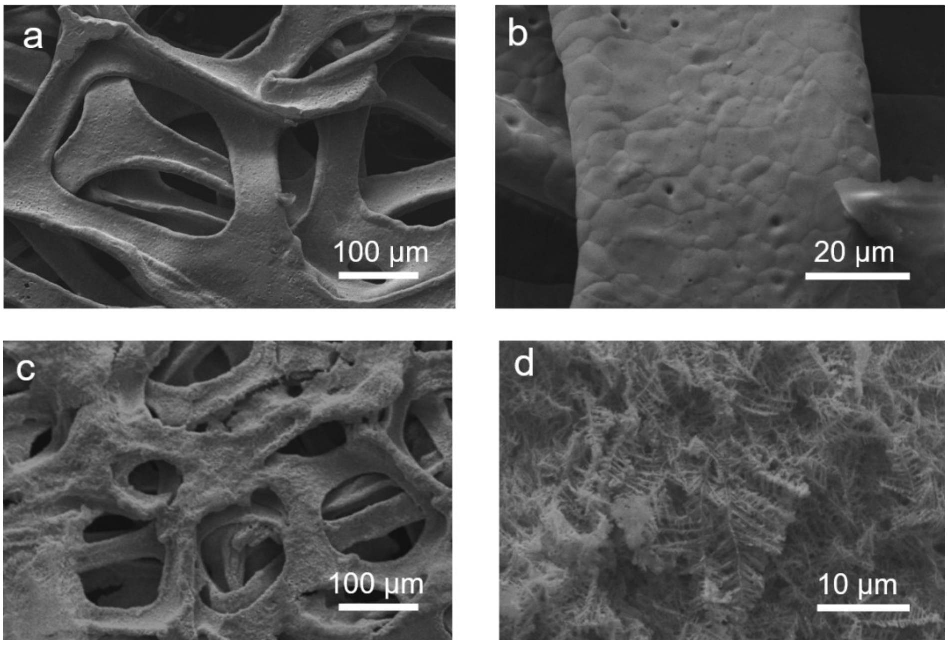

Figure S2 (a) SEM and (b) enlarge SEM images of Cu electrodes; (c) SEM and (d) enlarge SEM images of Cu@Bi.

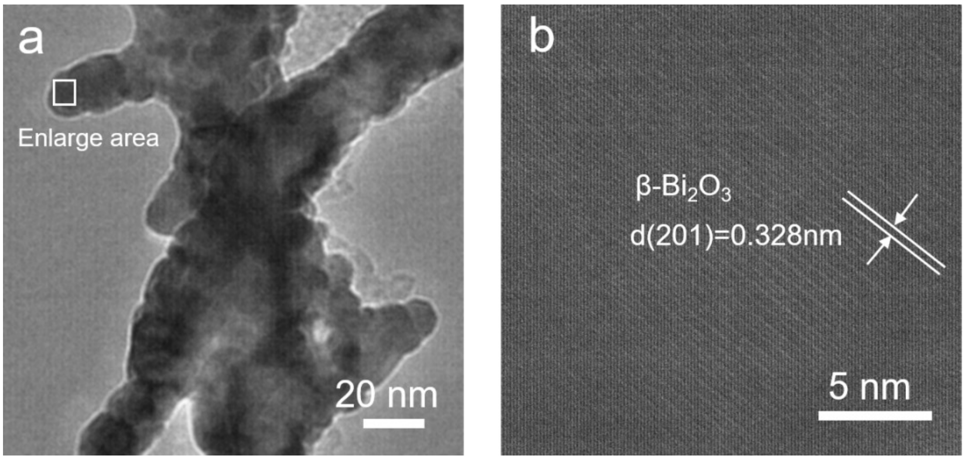

Figure S3 (a) TEM and (b) high resolution TEM image of Bi2O3 scraped from the Cu@BO electrode

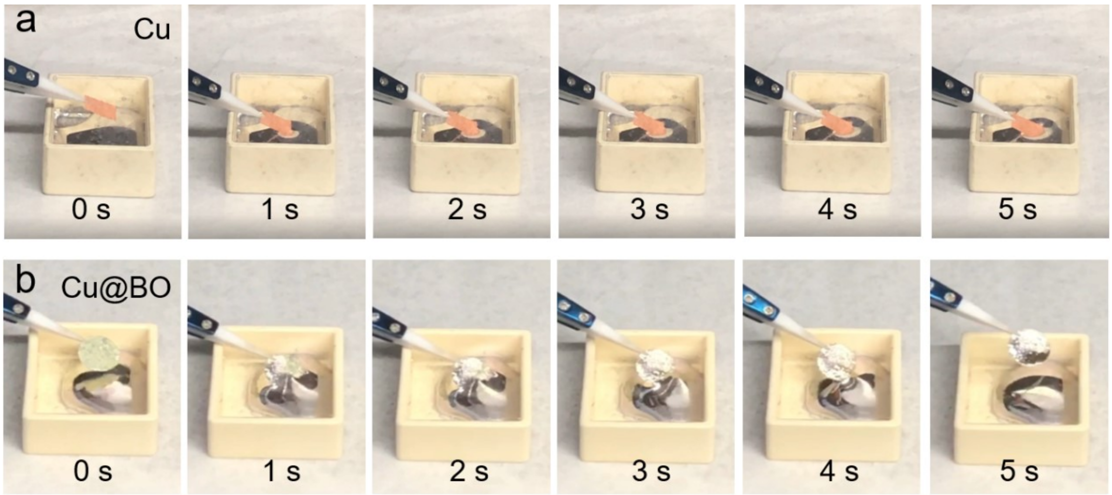

Figure S4 The potassiophilicity of the (a) Cu and (b) Cu@BO with molten K.

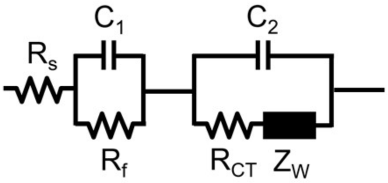

Figure S5 The corresponding equivalent circuit diagram of EIS.

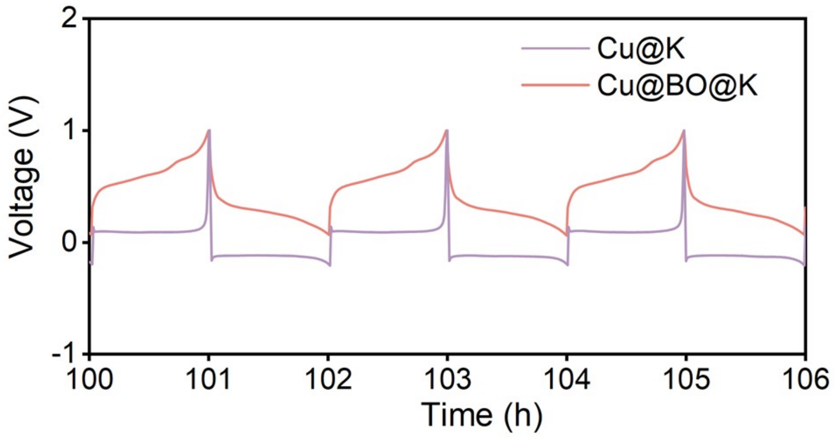

Figure S6 Charge-discharge curves of Cu||K and Cu@BO||K

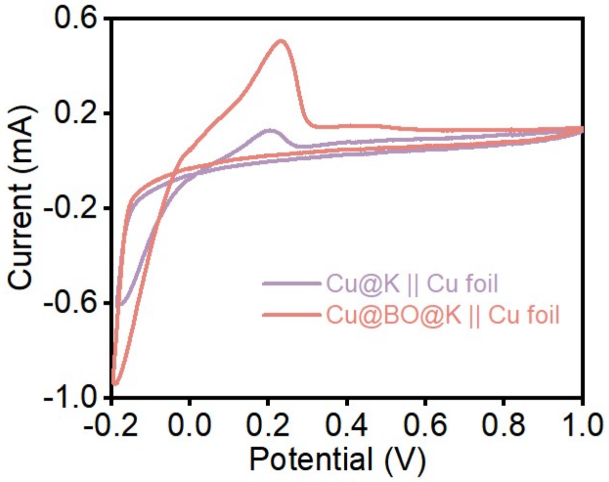

Figure S7 CV curves of Cu@K|| Cu, and Cu@BO@K|| Cu cells after 1 cycle

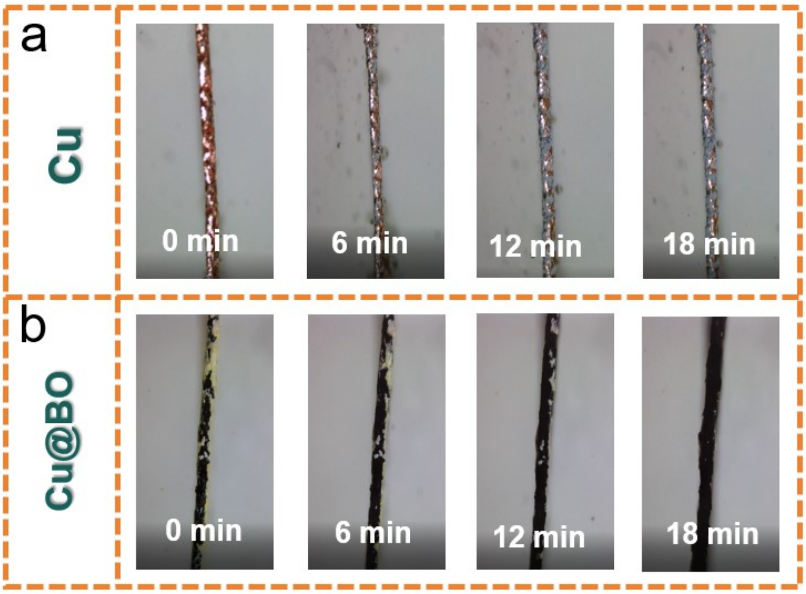

Figure S8 *In situ* optical microscope photographs for (a) Cu and (b) Cu@BO hosts at a plating areal current density of 10 mA cm^{-2}.

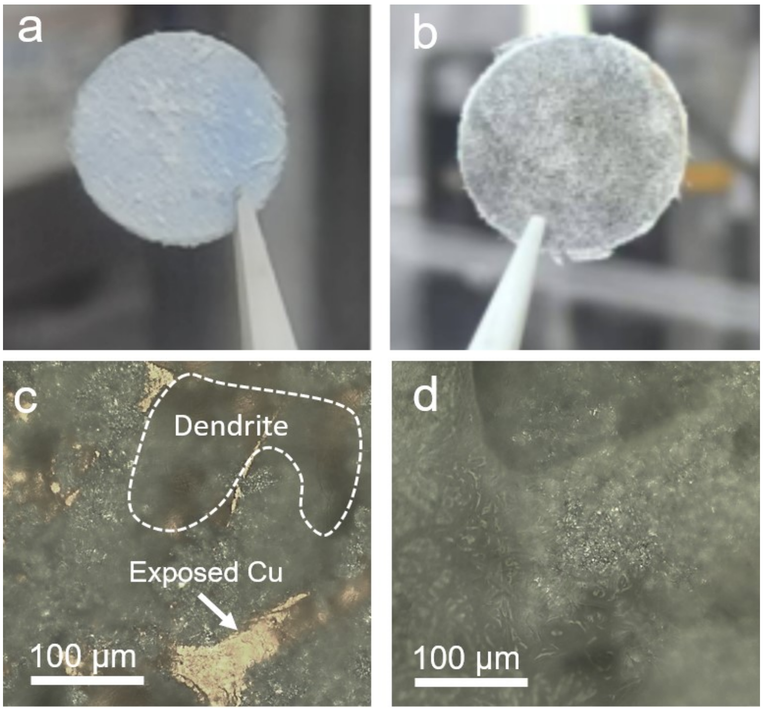

Figure S9 Photographs of the plated K metal on (a) Cu and (b) Cu@BO; High resolution optical microscope images of (c) Cu and (d) Cu@BO hosts after 10 cycles.

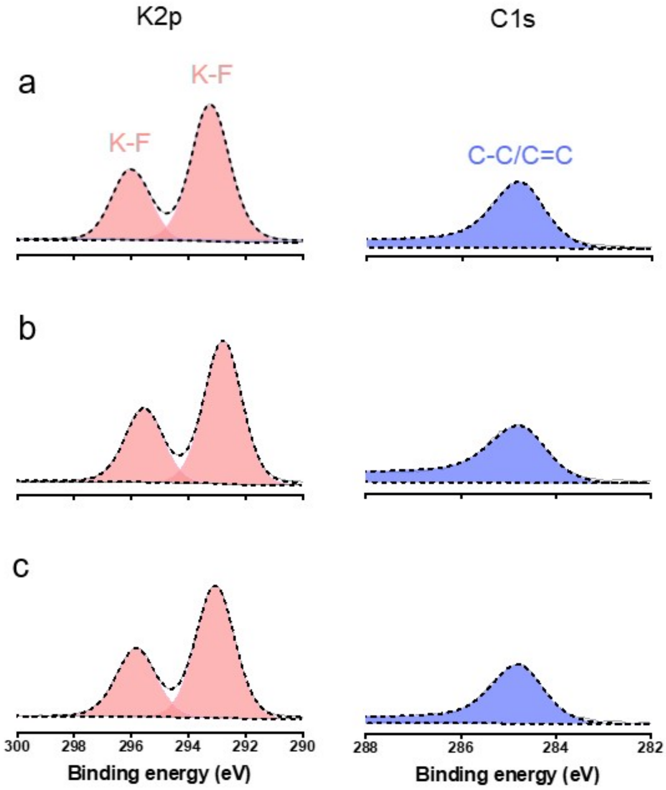

Figure S10 K 2p and C 1s XPS spectra of (a) bare K, (b) Cu@K and (c) Cu@BO@K after cycling for 2 h.

Reference

[1] T. Lu, F. Chen, J. Comput. Chem. 2012, 33, 580-592.

[2] J. Hutter, M. Iannuzzi, F. Schiffmann, et al., WIREs Comput. Mol. Sci. 2014, 4, 15-25.
